# Knowledge, sources and use of over-the-counter drugs by teachers in a Nigerian urban community

**DOI:** 10.4314/gmj.v59i3.5

**Published:** 2025-09

**Authors:** Chinonyerem O Iheanacho, Vincent Y Adam

**Affiliations:** 1 Department of Clinical Pharmacy and Public Health, Faculty of Pharmacy, University of Calabar, Calabar, Nigeria; 2 Department of Public Health and Community Health, School of Medicine, College of Medical Sciences, University of Benin, Benin City, Nigeria

**Keywords:** Over-the-counter (OTC) medicines, knowledge, secondary school teachers, sources, Nigeria

## Abstract

**Objectives:**

Responsible use of over-the-counter (OTC) drugs requires accurate knowledge of the drugs and when to seek professional help. The study assessed knowledge, sources and use of OTC medicines by secondary school teachers.

**Design:**

A descriptive cross-sectional survey was conducted using a multistage sampling method that involved systematic sampling.

**Setting:**

Ten secondary schools in Benin City, Nigeria

**Participants:**

Three hundred secondary school teachers who were either casual or permanent staff and who gave informed consent were included.

**Outcome measures:**

Respondents' knowledge of OTC drugs, their sources of OTC drugs, and the most commonly used OTC drugs, among the respondents.

**Results:**

Among the 300 study participants, the majority, 185 (61.7%), practised in public schools and more than a quarter, 82 (27.3%), had good knowledge of OTC drugs. Advertisement was the most frequently reported source of information 57(19.0%). Type of school (P=0.001), educational qualification (P=0.024) and reading of enclosed drug information leaflets (P=0.001) were significantly associated with level of knowledge. The major source of OTC medicines was pharmacies (158, 52.7%), and analgesics were mainly used (216, 72.0%), followed by vitamin supplements (172, 57.0%). Ability to recognise and treat minor ailments 132(44.0%,) was the major reason for OTC drug use.

**Conclusion:**

Poor knowledge of OTC medicines was common among the teachers; their major source of knowledge was advertisements, and analgesics were most used.

**Funding:**

None declared

## Introduction

Over-the-counter (OTC) drugs are widely used as dietary supplements and for other medical purposes,^1^ with their high availability resulting in high prevalence of use. Several sources have been reported to provide awareness on this class of drugs, and they include advertisements,^2^ pharmacists,^3^,^4^ and drug information leaflets,^5^,^6^, among others. OTC medicines are commonly available, easily accessible, and do not usually require a medical prescription for purchase. They are easily accessed from pharmacies, online stores and patent medicine stores. Also called non-prescription medicines, they are thought to be relatively safe even when used without medical supervision, usually as a result of a wide therapeutic index. Therefore, knowledge of OTC medicine by the users may be a major determinant of the general expectations from the medication and, subsequently, their use/misuse. They are mostly used in self-care, which involves the selection and use of OTC drugs in the management of self-recognised illnesses. Self-care is also defined as the primary public health resource within the healthcare system.^7^ Several specific reasons have been associated with its use, among which are adequate knowledge of the drugs, costs of treatment and minor ailments.^5^

Responsible self-medication requires good knowledge of drug indication, dosage, possible side effects, interactions, precautions, warnings, duration of use and when to seek professional help.^8^

Knowledge of OTC medications involves the ability to acquire and comprehend necessary information for appropriate use. OTC drug use patterns may vary among various populations, and socio-demographic factors, including educational level and age, among others, may influence them.^8^,^9^ Low to moderate awareness of OTC drugs has been reported in some populations^10^, however, this is not sufficient for the most appropriate use. Continuous education on the appropriate use of OTC drugs in a population has been identified as a major way to equip the population with the relevant knowledge for good outcomes.^11^ However, poor attention has been given to the knowledge of OTC medicines among teachers, while the majority of the studies have targeted students, health professionals and the general public.^1^,^4^,^5^,^6^,^8^ Meanwhile, teachers are gateways to the provision of education to the larger population.

In Nigeria, OTC drugs are available in various forms and places, including in open marketplaces and commercial vehicles. This allows almost unlimited access to them from marketers who may not have sufficient and accurate information to offer the consumers. This may leave the consumers with little or no reliable information on the OTC drug being used, which poses the risk of misinformation and misuse. It is therefore imperative to understand the knowledge base, as well as its sources and use, to elucidate the need for targeted educational interventions. Secondary school teachers are educational elites and strategically positioned to transfer knowledge to teenagers and young adults, suggesting their suitability for the study. This study assessed the knowledge, sources and use of OTC drugs, with a view to providing insight and understanding of their potential or associated safety. This is expected to guide targeted interventional approaches.

## Methods

A descriptive cross-sectional study was conducted among 300 secondary school teachers in Egor Local Government Area (LGA), Benin City, Nigeria. The study setting is a metropolitan city that comprises several primary, secondary, and tertiary educational institutions, as well as health facilities, including pharmacies. Public and private secondary school teachers who were either casual or permanent staff and who gave informed consent were included in the study, but teachers who were absent at the time of the study were excluded. The total number of secondary school teachers was obtained from the secondary education board, and this constituted the study population. The total study population was 452, and the sample size for the study was calculated using the online sample size calculator (Calculator.net).^12^ This was done by using a 5% error margin, 95% confidence interval and a population proportion of 50%, which yielded a sample size of 208. This number was rounded up to 300 to increase the effect size and minimise statistical noise between study centres, which mostly arises from variations in the participants' characteristics and associated with multicenter studies.^13^,^14^

A multistage sampling method was used in conducting the study. Stage 1 was carried out using a simple random sampling method through balloting. This was used to select 5 out of 10 wards in the LGA for the research. Stage 2 was also conducted using a simple random sampling method, from which one private and one public secondary school were selected from each of the selected wards. Stage 3 involved a systematic sampling method using probability proportional to size (PPS) to select study respondents. PPS was done to determine the actual number of teachers to be drawn from each school using a sampling fraction. The systematic sampling was done to select the teachers who would participate in the study from the lists of teachers in the selected public and private schools using a sample interval. This approach was adopted to obtain a randomised sample.

The multistage sampling technique in this study involved two stages: proportional and systematic random sampling methods. The first stage- proportional sampling was employed to ensure a proportional number of included private and public secondary schools. The systematic random sampling technique was preferred for the selected schools to ensure the randomisation of the sample (in each school). Findings from a randomised sample are usually more representative than a convenient sample.

The data collection tool was a structured, self-administered questionnaire comprising three sections. It was validated through pretesting among twenty respondents of similar demographics and expert assessment (face validity). Section one was used to collect respondents' information on socio-demographics, while section two was used to assess respondents' knowledge and sources of information on OTC drugs. Section three assessed the sources and use of OTC drugs.

Knowledge of OTC medicines was assessed using five questions that had a yes/no response scale. Responses on knowledge were scored to make a clear demarcation of respondents' knowledge level on OTC drugs. The minimum score was “1” while the maximum was “5”. The correct answer was assigned “1” mark, and a wrong answer attracted “0”. To further discern the knowledge differences in the groups, knowledge was further divided into three: poor knowledge [0-2], fair knowledge [3] and good knowledge [4-5] on OTC drugs. This was used to assess the respondents' knowledge.

Study outcome measures were respondents' knowledge of OTC drugs, their sources of OTC drugs, and the most commonly used OTC drugs.

Statistical analysis was done using IBM SPSS version 21.0 statistical software. Means for age and duration of practice were tested, and the frequency of OTC drug use was also determined. Chi-square test was used to test for associations (P < 0.05).

Ethical approval was obtained from the Ethics and Research Committee of the University of Benin Teaching Hospital, and its protocol number was ADM/E22/A/VOL.VII/1284. Institutional approval and individual informed consent were also obtained prior to the study. Health education on the rational use of OTC drugs was also given at the end of data collection in each school to the members of staff and students.

## Results

The majority of respondents were in the 25-40 years age group. The mean (standard deviation) age and duration of practice for male and female respondents were: age [36.0(±11.4) for males and 34.9(±11.9) for females], and duration of practice was 9.9(±9.9) for males and 8.4(±7.9) for females. More than half of the respondents, 186 (62.0%), were females, 191 (63.7%) had a bachelor's degree, and 167 (55.7%) had practised teaching for five years respectively. The majority, 185 (61.7%), practised in public schools. See Table 1.

**Table 1:** Socio-demographics of respondents. N = 300

Variables	Frequency n (%)
**Age (years)**	
**<25**	94 (31.3)
**25 -40**	120 (40.0)
**>40**	86(28.7)
**Sex**	
**Male**	114(38.0)
**Female**	186(62.0)
**Educational qualification**	
**Postgraduate**	43(14.3)
**Bachelors' degree**	191(63.7)
**Diploma**	25(8.3)
**Less than diploma**	41(13.7)
**Type of school**	
**Public**	185(61.7)
**Private**	115(38.3)
**Duration of practice**	
**<5**	167(55.7)
**6 -15**	76(25.3)
**16 – 25**	28(9.3)
**>25**	29(9.7)
**Marital status**	
**Never married**	151(50.3)
**Ever married**	149(49.7)

Slightly over a quarter of the respondents, 82(27.3%), had good knowledge of OTC drugs. About a fifth, 57 (19.0%) and 49 (16.3%) got their information about OTC drugs from advertisements and pharmacists, respectively. Almost 10.0% of the respondents got information about OTC drugs from the school and family members. Physicians were the least source of information on OTC drugs, 17 (5.0%). This is shown in Table 2.

**Table 2:** Respondents' sources of information and knowledge of OTC drugs. N = 300

Variables	Frequency n(%)
**Sources of awareness on OTC drug**	
**Advertisement**	57 (19)
**Pharmacist**	49(16.3)
**Family**	36(12.0)
**School**	32(10.7)
**Friends**	27(9.0)
**Medical Doctor**	17(5.7)
Others*	12(4.0)
**Level of knowledge**	
**Poor**	82 (27.3)
**Fair**	136(45.4)
**Good**	82(27.3)

* Others – internet, patent medicine vendors and nurse

Table 3 shows that the most commonly used OTC drugs were analgesics, 216 (72.0%), followed by vitamin supplements, 172(57.0%). Analgesics were taken at least monthly by almost half of the respondents, 99(45.8%). Other OTC drugs used by the participants were cough mixtures (20.7%) and ointments (15.0%), among others, at varying frequencies. The reasons for usage and sources of patronage for OTC drugs are also shown in Table 3.

**Table 3:** Sources, reason for purchase and frequency of use of OTC medicines

Variables	Frequency (n)%
**Place of Purchase 300(100%)**	
**Pharmacy**	158(52.7)
**Patent medicine store**	128(42.7)
**Others^+^**	4(4.7)
**Reasons for purchase 300(100%)**	
**Ability to treat minor ailments**	132(44.0)
**It saves time**	88(29.3)
**Less expensive**	71(23.7)
*Others	9(3.0)
**Frequency of use**	
**Analgesics 216(72.0%)**	
**At least daily**	21(9.7)
**At least weekly**	33(15.3)
**At least monthly**	99(45.8)
**Seldom**	63(29.2)
**Vitamin Supplements: 172(57.0%)**	
**At least daily**	30 (17.4)
**At least weekly**	67(39.0)
**At least monthly**	54(31.4)
**Seldom**	1(12.2)
**Cough mixtures: 62(20.7%)**	
**At least daily**	1(1.6)
**At least weekly**	6(9.7)
**At least monthly**	22(35.5)
**Seldom**	33(53.2)
**Ointments: 45(15.0%)**	
**At least daily**	9(20.0)
**At least weekly**	11(24.4)
**At least monthly**	18(40.0)
**Seldom**	7(15.6)
**^+^Other drugs 14(4.7%)**	
**At least daily**	1(7.1)
**At least weekly**	1(7.1)
**At least monthly**	4(28.6)
**Seldom**	8(57.1)

+ Others = antimalarial medicines and antibiotics

* Others = easily accessible and those who reported that they do not take medicines.

Others^+^ = markets and gifts

There was a significant difference in the OTC knowledge score of private school teachers and the public school teachers (P = 0.001). Other socio-demographics such as age, sex, marital status, educational qualification and duration of practice were not statistically significantly associated with knowledge of OTC drugs. The level of knowledge of OTC drugs was also significantly related to the type of school and the reading of OTC drug leaflets by the teachers (P = 0.001), respectively.

Almost half, 51(46.4%) of the respondents who read their OTC drugs leaflets “always” had fair knowledge on OTC drugs. Half, 79(50.0 %) of those who read “sometimes” had fair knowledge, while less than three-quarters, 23(71.9 %) of those who “never read” had poor knowledge. The relationship was statistically significant (P = 0.001). See Table 4.

**Table 4:** Respondents' characteristics and knowledge on OTC drugs

Variables	Knowledge of OTC drug			

	Poor n = 82 (%)	Fair n = 136(%)	Good n = 82 (%)	P - value
**Age (years)**				
**<25**	22 (23.4)	36 (38.3)	36 (38.3)	
**25 -40**	34 (28.3)	58 (48.3)	28 (23.3)	.076
**>40**	26 (30.2)	42 (48.8)	18 (20.9)	
**Sex**				
**Male**	32 (27.4)	53 (46.9)	29 (25.7)	
**Female**	50 (26.9)	83 (44.6)	53 (28.5)	0.564
**Educational qualification**				
**Less than diploma**	15 (36.6)	19 (46.3)	7 (17.1)	
**Diploma**	4 (16.0)	10 (40.0)	11 (44.0)	
**Bachelors' degree**	52 (27.2)	85 (44.5)	54 (28.3)	.280
**Postgraduate**	11 (25.6)	22 (51.2)	10 (23.3)	
**Type of school**				
**Public**	63 (34.1)	83 (44.9)	39 (21.1)	
**Private**	19 (16.5)	53(46.1)	43 (37.4)	0.001*
**Duration of practice**				
**<5**	38 (22.8)	74 (44.3)	55 (32.9)	
**6 -15**	24 (31.6)	36 (47.4)	16 (21.1)	0.125
**16 – 25**	12 (42.9)	10 (35.7)	6 (21.4)	
**>25**	8 (27.6)	16 (55.2)	5 (17.2)	
**Marital status**				
**Never married**	36 (23.8)	66 (43.7)	49 (32.5)	
**Ever married**	46 (30.9)	70 (47.0)	33 (22.1)	0.108
**Read enclosed drug information leaflets**				
**Always**	20 (18.2)	51 (46.4)	39 (35.5)	
**Sometimes**	39 (24.7)	79 (50.0)	40 (25.3)	0.001*
**Never**	3 (71.9)	6 (18.8)	3 (9.4)	

Educational qualification was significantly associated with awareness of the presence of side effects in OTC medicines P = 0.024). Respondents who had less than a diploma degree agreed least to the presence of side effects in these medicines. The type of school was also significantly associated with this awareness (P = 0.006), with respondents in private schools having a higher proportion of 98 (85.2%) than those in public schools 132 (71.4%), of the respondents who were aware of the presence of side effects in OTC drugs. Other associations are shown in Table 5.

**Table 5:** Socio-demographics and awareness of the presence of side effects in OTC drugs

Variables	Presence of side effects in OTC drugs		

	Yes n = 230 (%)	No n = 70 (%)	P-value
**Age (years)**			
**<25**	76 (80.9)	18 (19.1)	
**25 -40**	92 (76.7)	28 (23.3)	.382
**>40**	2 (72.1)	24 (27.9)	
**Sex**			
**Male**	88 (77.9)	25 (22.1)	
**Female**	142 (76.3)	44 (23.7)	0.184
**Educational qualification**			
**Less than diploma**	25 (61.0)	16 (39.0)	
**Diploma**	23 (92.0)	2 (8.0)	0.024*
**Bachelors' degree**	147 (77.0)	44 (23.0)	
**Postgraduate**	35 (81.4)	8 (18.6)	
**Type of school**			
**Public**	132 (71.4)	53 (28.6)	.006*
**Private**	98 (85.2)	17 (14.8)	
**Duration of practice**			
**<5**	133 (79.6)	34 (20.4)	
**6 -15**	57 (75.0)	19 (25.0)	0.484
**16 – 25**	19 (67.9)	9 (32.1)	
**>25**	21 (72.4)	8 (27.6)	
**Marital status**			
**Never married**	118 (78.1)	33 (21.9)	
**Ever married**	112 (75.2)	37 (24.8)	0.542

* Significant

## Discussion

Many respondents did not have good knowledge of OTC drugs, and most obtained information regarding their OTC drugs through advertisements. Although knowledge of OTC drugs was low, our study found that respondents who worked in private schools had better knowledge of OTC drugs, compared to others. Analgesics were the most commonly used OTC medicines in this study, followed by vitamin supplements. The major reason for the use of OTC drugs, as reported in this study, was the ability to recognise and treat minor ailments. Over half of the respondents got their OTC drugs from pharmacies.

Only slightly above a quarter of the respondents had good knowledge of OTC drugs; this finding corroborates findings in a previous study conducted in South-East Nigeria, where most of the OTC medicine users were reported to rely on past experiences rather than professional information to arrive at the dose to be administered to their children.^15^ Similarly, insufficient knowledge of OTC medicines was observed in previous studies in the United States of America,^16^ and Poland.^17^ The finding of this study is also consistent with reports from a similar study in Sweden, where the study population did consider OTC drugs to be harmless,^18^ and another study where the majority of the population were unaware of the indications for use of vitamin supplements.^19^ This was, however, different in another study where the majority of the respondents had good knowledge of OTC drugs.^9^ Low or inadequate counselling on medicine use may be a major contributor to this observed insufficient knowledge. Poor knowledge of medicines by users could result in inappropriate use and hence adverse drug reactions (ADRs), some of which may be life-threatening. Poor knowledge of OTC drugs predisposed users to risky practices in the use of OTC drugs.^20^ Drug information units should therefore be functional and effective, as this will promote knowledge on these drugs.

Advertisement was the most common source of information on OTC drugs for the respondents, and this is reflective of the widespread use of media for publicity. This finding is consistent with the report from a study conducted in Pakistan, where advertisement was a major source of information on OTC drugs.^21^ Advertisements create awareness of various products and may not be a good source of knowledge as they merely promote products where benefits may be exaggerated and associated risks downplayed. However, this finding may buttress the need to use advertisement platforms such as the electronic and print media as sources for disseminating vital information, including the rational use of OTC medicines to the public. Therefore, sections should be created in widely read newspapers or television/radio programmes to educate the public on the safe use of OTC drugs. Meanwhile, this finding differs from the reports of a study conducted in India on awareness and attitude towards OTC drugs, where advertisement was a major source for only a few respondents.^2^ More than half of the respondents in Egypt reported having their OTC drug information from the pharmacists.^3^ Another study also reports the pharmacist as the major information source for its respondents in India.^4^ This difference in findings could be associated with differences in the socioeconomic background of the people, beliefs and availability and accessibility of media sources. Findings also suggest that the accessibility of pharmacists is essential in the dissemination of OTC medicine information and should be encouraged.

Although good knowledge of OTC drugs was low, it was found to be predominant in the respondents who practised in private schools. Awareness of potential drug-drug interactions in OTC drugs was also predominantly found in respondents who practised in private schools and those who had never been married. In another study, Educational level, occupation and knowledge of OTC drugs significantly affected risky practice, with lower educational status presenting higher risks.^20^ Another study also identified a significant association between knowledge of OTC drugs and age, marital status and education.^9^ A significant number of respondents in a study had low awareness of the side effects of OTC drugs.^4^ Good knowledge of OTC medicines empowers persons for safer and rational use of the medicines.

Analgesics, followed by vitamin supplements, were the predominantly used OTC medicines in this study. Analgesics were also reported as the most commonly used OTC drugs in a similar study.^22^ In another study on self-medication conducted in Malaysia, analgesics were reported to be the most widely used medicines after vitamin supplements.^23^ Similarly, analgesics were reported to be the most used in Eritrea,^20^ in South West Nigeria,^24^ and in Sweden.^19^ Analgesics have also been reported to be used in self-harm in overdose in certain patients, calling for closer focus of health professionals, especially pharmacists, on regular, effective medicine education.^25^ The anti-inflammatory and antipyretic properties of analgesics could account for their widespread use, as chronic musculoskeletal pain seems to constitute a high global burden, especially with an increase in age. Chronic use of analgesics, particularly the Non-steroidal Anti-inflammatory Drugs (NSAIDs), is, however, associated with several risk factors. One of these is the increased risk of end-stage renal disease in patients with nephrotic disorders as a result of its nephrotoxicity. Gastrointestinal complications such as peptic ulcer disease (PUD) are also well recognised risks of NSAIDs, especially in chronic use. Also, Aspirin, having anticoagulant properties, increases the risks of bleeding in chronic use, especially when taken concomitantly with other anti-thrombotic drugs like warfarin. Therefore, persons with a need for long-term analgesics should seek medical help.

The major reason for the use of OTC drugs, as reported in this study, was the ability to recognise and treat minor ailments. The respondents' high educational level could account for their confidence in this ability. Meanwhile, previous studies in Nigeria have noted high prevalence of OTC misuse^26^ and the misuse of antibiotics following their OTC availability in Nigeria.^27^,^28^ Therefore, preventive and educational programs on self-care conducted regularly could help in enhancing this skill in OTC medicine users. Cost was the reason for use as reported by the majority in a study conducted in Malaysia.^4^,^29^ The difference in level of awareness and economic status of the respondents may have resulted in the difference in self-medication with OTC medicines, without appropriate skills, which may result in increased morbidity and mortality. Measures should be put in place to make professional health care accessible in terms of cost, to ensure adequate utilisation when needed.

Over half of the respondents got their OTC drugs from pharmacies. A previous study showed good knowledge and a positive attitude of pharmacists towards drug information services.^30^ Therefore, this finding is a commendable pattern and should be encouraged and sustained, as it provides an opportunity for appropriate counselling and OTC drug information by pharmacists to the users during purchase, as noted in a previous study.^31^ A study in Mexico reported similar findings.^28^ This was also similar to another study in Malaysia, where the OTC medicines were bought from pharmacies.^32^ Confidence in getting the appropriate drug and relevant information could account for the high patronage. Emphasis should always be made to consumers on the need to seek counsel regarding their OTC medicines.

The systematic sampling design adopted in this study enhances the generalisation of study findings. However, some limitations were noted to be associated with this study, including the risk of self-reporting bias. This follows the self-reporting approach of assessment employed in the study.

## Conclusion

Poor knowledge of OTC medicines was common among secondary school teachers in Benin City, and their major source of knowledge was advertisements, while pharmacies were the major source of the OTC drugs. Analgesics were the predominantly used OTC medicines by secondary school teachers. This underscores the need for medication education at the point of OTC drugs purchase in pharmacies. Pharmaceutical companies in their drug advertisements should carry out routine public health enlightenment to educate the populace on safe use of OTC medicines through electronic media.

## Figures and Tables

**Figure 1:**
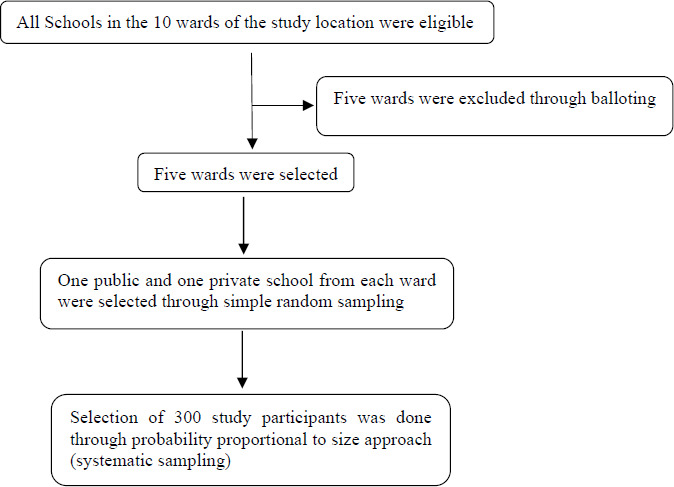
Flow chart showing details of sampling technique
